# Aquaglyceroporin PbAQP is required for efficient progression through the liver stage of *Plasmodium* infection

**DOI:** 10.1038/s41598-017-18987-3

**Published:** 2018-01-12

**Authors:** Dominique Promeneur, Godfree Mlambo, Peter Agre, Isabelle Coppens

**Affiliations:** Department of Molecular Microbiology and Immunology Johns Hopkins Malaria Research Institute, Johns Hopkins University Bloomberg School of Public Health, Baltimore, 21205 MD USA

## Abstract

The discovery of aquaglyceroporins (AQP) has highlighted a new mechanism of membrane solute transport that may hold therapeutic potential for controlling parasitic infections, including malaria. *Plasmodium* parasites express a single AQP at the plasma membrane that functions as a channel for water, nutrients and waste into and out cells. We previously demonstrated that *Plasmodium berghei* targeted for *PbAQP* deletion are deficient in glycerol import and less virulent than wild-type parasites during the blood developmental stage. Here, we have examined the contribution of PbAQP to the infectivity of *P*. *berghei* in the liver. *PbAQP* is expressed in the sporozoite mosquito stage and is detected at low levels in intrahepatic parasites at the onset of hepatocyte infection. As the parasites progress to late hepatic stages, *PbAQP* transcription increases and PbAQP localizes to the plasma membrane of hepatic merozoites. Compared to wild-type parasites, PbAQP-null sporozoites exhibit a delay in blood stage infection due to slower replication in hepatocytes, resulting in retardation of merosome production. Furthermore, *PbAQP* disruption results in a significant reduction in erythrocyte infectivity by hepatocyte-derived merozoites. Hepatic merozoites incorporate exogenous glycerol into glycerophospholipids and PbAQP-null merozoites contain less phosphatidylcholine than wild-type merozoites, underlining the contribution of *Plasmodium* AQP to phospholipid syntheses.

## Introduction

Malaria is due to protozoan parasites of the genus *Plasmodium*, and this parasitic infection causes a global health epidemic with considerable morbidity and ∼429,000 deaths in 2015 according to WHO (www.who.int/gho/malaria). Among species that infect humans, *Plasmodium falciparum* is found worldwide in tropical and subtropical areas and is the deadliest *Plasmodium* species, causing cerebral malaria associated with anemia, coma and death primarily among young children. After infection into the skin through the bite of an *Anopheles* mosquito, the parasite (sporozoite form) reaches a blood vessel and is transported to the liver^1^ wherein it actively invades hepatocytes by forming a parasitophorous vacuole (PV)^2^. Intrahepatic *Plasmodia* undergo asexual reproduction (schizont liver form), then bud off from hepatocytes as membrane-bound structures (merosomes) containing thousands of infectious parasites (hepatic merozoite forms) that are proficient to invade erythrocytes where additional rounds of asexual replication occur.

Transmembrane import of nutrients and export of toxic products are paramount for all organisms. *Plasmodium* parasites express several transport proteins that facilitate the exchange of molecules across membranes, e.g., the PV membrane, the plasma membrane and organellar membranes, and these transporters are essential to sustain malaria infection^3^. A single multifunctional aquaglyceroporin channeling selective substrates is expressed by *P. falciparum* (PfAQP) and by the rodent parasite *Plasmodium berghei* (PbAQP) at the plasma membrane where it mediates the passage of glycerol, water, urea, small polyols and carbonyl compounds through its amphipathic pore^4–6^.

Additionally, PfAQP facilitates the expulsion of residual cytotoxic byproducts such as ammonia resulting from the conversion of amino acids to α-ketoacids, and carbonyl compounds from glycolysis, which preserves the parasite from self-intoxication^7,8^. The directionality of water and solute diffusion via AQP is determined by the prevailing osmotic and chemical gradients^9^. Obviously, the functions of PfAQP and PbAQP are crucial for malaria parasites as they face drastic osmotic changes during host tissue migration. Due to their rapid growth, *Plasmodium* sp. are engaged in the massive biosynthesis of glycerolipids, for which they require exogenous glycerol, to satisfy their rapid growth both in the liver and the blood. Thus, aquaporins and aquaglyceroporins broadly function in water osmoregulation, lipid syntheses, oxidative stress, energy production and carbon/nitrogen balance. Interestingly, PfAQP is also permeant to antiparasitic compounds such as metalloids, e.g., arsenites or antimonials, revealing the potential for novel chemotherapeutic approaches based on the exploitation of *Plasmodium* AQP for delivering therapeutics into the parasite cell^10^. Genetic ablation of *PbAQP* in *P. berghei* results in deficiency in glycerol acquisition by blood forms^5^. Furthermore, PbAQP-null parasites proliferate about half as fast as wild-type (WT) parasites and are less virulent in mice.

So far, the function and localization of *Plasmodium* AQP as well as its contribution to parasite development have been only investigated for the malaria asexual blood stage. In this study, we focus on the expression and physiological relevance of PbAQP during the liver developmental stage. Using the *P. berghei* rodent malaria model, we showed that PbAQP is predominantly expressed during late stage development in hepatocytes. By exploiting PbAQP-null parasites^5^, we determined that PbAQP is required for efficient progression through the liver stage of infection and phospholipid synthesis in hepatic merozoites.

## Results

### *P. berghei* aquaglyceroporin is expressed during the liver developmental stage

**Figure 1:**
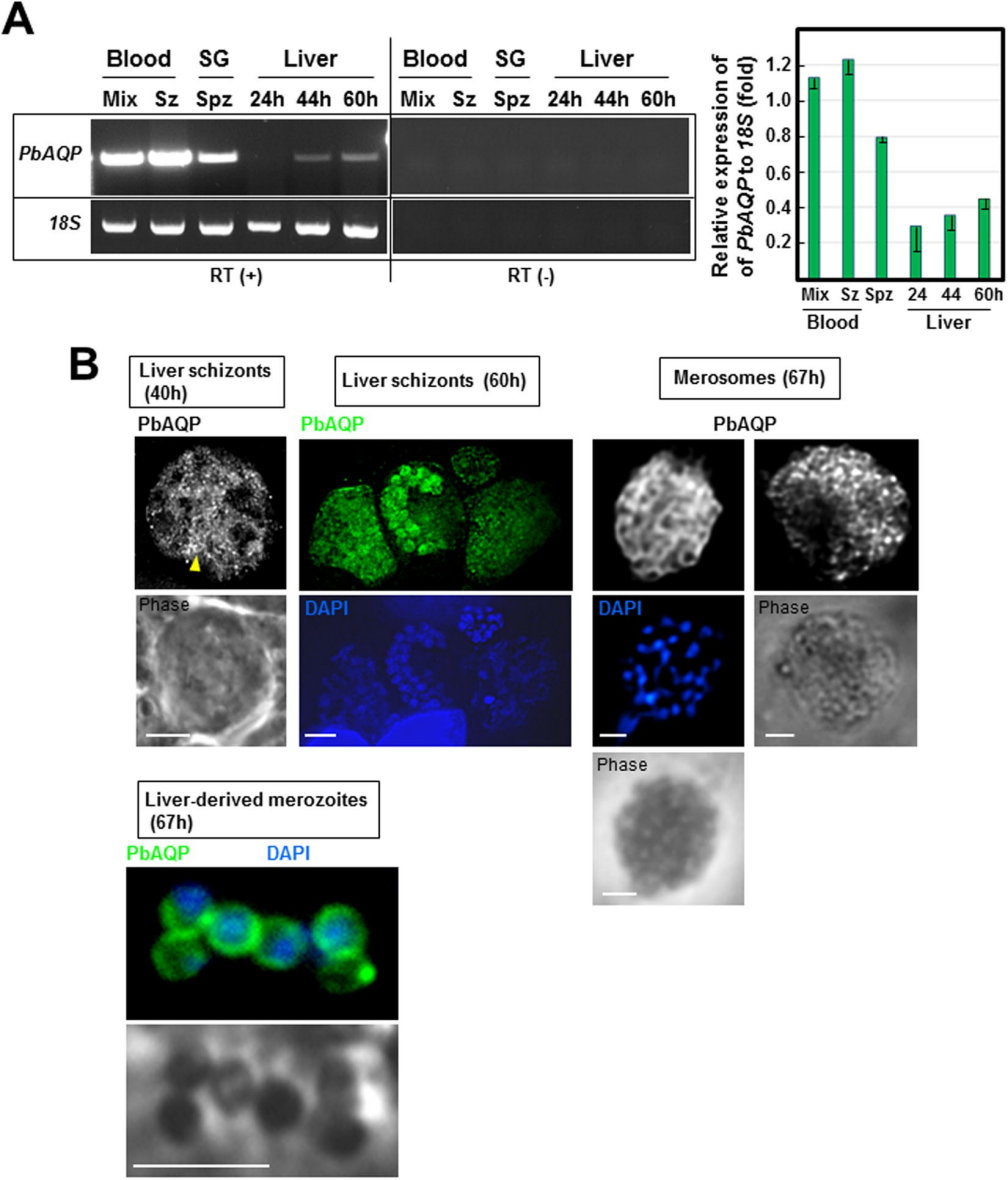
Expression of *PbAQP* during *P. berghei* developmental stages and localization of PbAQP in liver forms. (**A**) Transcriptional profiles of PbAQP in mixed blood forms, schizonts (Sz), salivary glands (SG) sporozoites (Spz) and liver forms at 24 h, 44 h and 60 h post-infection of Hepa1-6 cells assayed by RT-PCR. To verify the absence of genomic DNA contamination, RT-PCR reactions were set up without reverse transcriptase (RT) detecting no band (-). Two separate representative gels with cropped bands of PCR products for either *PbAQP* or *18S rRNA* (loading control) are shown. The products of RT-PCR reactions with or without RT were run on the same gel but at different area to avoid contamination. The gel have been cut and the assembled products of RT-PCR with or without RT are separated by a vertical line. The 18S rRNA gene was used as a control to quantify PbAQP transcripts throughout the developmental stages of the parasite. Two separate agarose gels have been run for each transcripts The graph shows the quantitative analysis of *PbAQP* expression relative to *18S rRNA* from 3 independent experiments. (**B**) Localization of PbAQP in intrahepatic *P. berghei* 40 h and 60 h p.i., merosomes and hepatic merozoites liberated from merosomes 67 h p.i. by IFA using anti-PbAQP antibodies. Arrowhead points to the tubular profiles. DAPI, blue. Bars, 10 μm.

As a first approach to investigate the importance of PbAQP for the liver stage, we examined the transcriptional profiles of *PbAQP* during parasite development in liver cells from 24 h to 60 h, which corresponds to the duration of the intrahepatic development of *P. berghei*. In contrast to strong expression of the PbAQP gene in mixed blood forms, blood schizonts, and to a lesser extent in sporozoites, *PbAQP* transcript levels were very low in liver forms at 24 h post-infection (p.i.), and subsequently, *PbAQP* transcription increased during *Plasmodium* schizogony in hepatocytes (Fig. 1A).

To verify the expression of PbAQP at the protein level, we performed immunofluorescence assays (IFA) on intrahepatic forms of *P. berghei* (schizonts, merosomes and hepatic merozoites), using anti-PbAQP antibodies we previously generated and used to reveal the localization of PbAQP on the plasma membrane of blood forms^5^. At 40 h p.i. of liver cells, the PbAQP pattern was largely diffuse in the parasite’s cytoplasm and sometimes associated with tubular structures (Fig. 1B, arrowhead). After an initial growth phase associated with karyokinesis, the parasite membrane begins to invaginate ~60 h p.i. and wraps around individual hepatic merozoite buds to form mature hepatic merozoites within the PV, then inside merosomes^10,11^. At this time, the localization of PbAQP became more peripheral, delimiting individual parasites identified by DAPI, suggesting a labeling of the parasite’s plasma membrane. The peripheral staining pattern was also observed in intramerosomal parasites, as illustrated for two merosomes detached from the host cell monolayer. Finally, merozoites, which were mechanically liberated from merosomes, expressed PbAQP on the plasma membrane.

### PbAQP-null sporozoites have a delayed prepatent period compared to wild-type sporozoites

**Figure 2:**
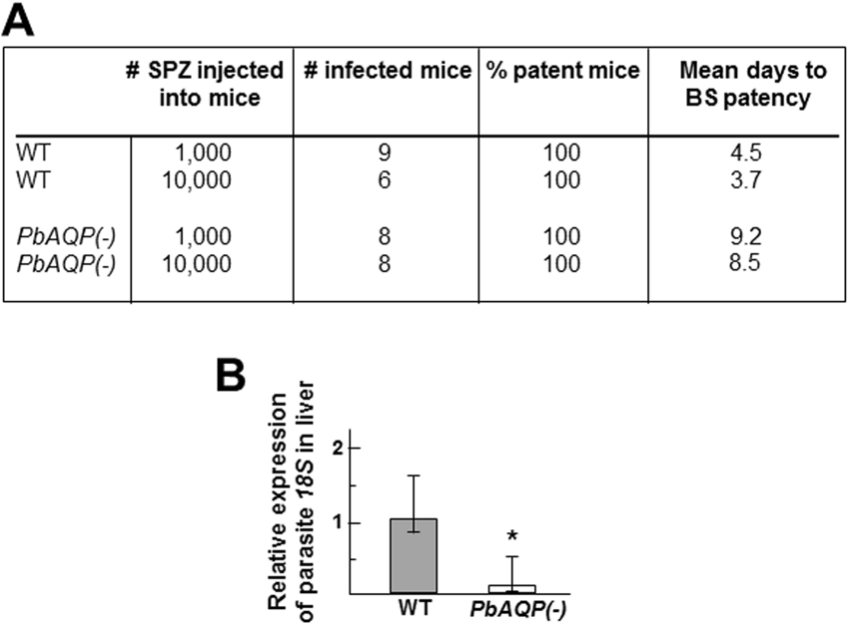
Comparison of the virulence of WT and PbAQP-null sporozoites and liver infectivity in mice. (**A**) Infectivity of mice with WT and PbAQP-null *P. berghei* (*PbAQP*(-)) sporozoites monitored by blood stage (BS) patency. Swiss-Webster mice were infected with 1,000 or 10,000 sporozoites by i.v. and blood-stage patency was monitored daily by evaluation of Giemsa-stained blood smears from day 2 to day 14 post sporozoite infection. Data are mean days ± S.D. (**B**) Mice liver load with WT and PbAQP(-) liver forms assessed 44 h after i.v. injection of 4,500 sporozoites. Livers from 6 to 9 Swiss-Webster mice were collected for RNA extraction to monitor *P. berghei 18S rRNA* transcript levels by quantitative real-time PCR. Data are means ± S.D. *p < 0.045.

We next investigated the infectivity of PbAQP-null sporozoites by inoculating these knockout parasites and wild-type (WT) controls intravenously (i.v.) into mice and determining the prepatent period (the time elapsed from the inoculation of sporozoites until the emergence of parasites in the blood) by Giemsa-stained blood smears. In mice, an i.v. inoculum of 1,000 WT sporozoites typically results in detectable blood stage parasites at a prepatent period of three to five days. Following inoculation of 1,000 or 10,000 PbAQP-null sporozoites into Swiss-Webster mice, all animals developed parasitemia (Fig. 2A). However, the prepatent period of knockout sporozoites was consistently delayed by approximately five days as compared to mice inoculated with WT sporozoites.

Given the delay in the prepatent period after injection of PbAQP-null sporozoites compared to WT parasites, we investigated sporozoite infection *in vivo*. To measure the parasite load in livers, mice were infected by i.v. with 4,500 WT or PbAQP-null sporozoites, and their livers were harvested after 55 h of infection to assess parasite liver burden by monitoring the expression of the *P. berghei* 18S rRNA gene (Fig. 2B). Significantly lower levels of 18S rRNA transcripts were observed in livers infected with PbAQP-null parasites, in comparison to WT parasites. The delay in knockout parasite development in the liver suggests a requirement for PbAQP in the optimal infectivity of *P*. *berghei* in mammalian livers.

### PbAQP-null sporozoites invade hepatocytes normally but replicate more slowly than wild-type parasites

**Figure 3:**
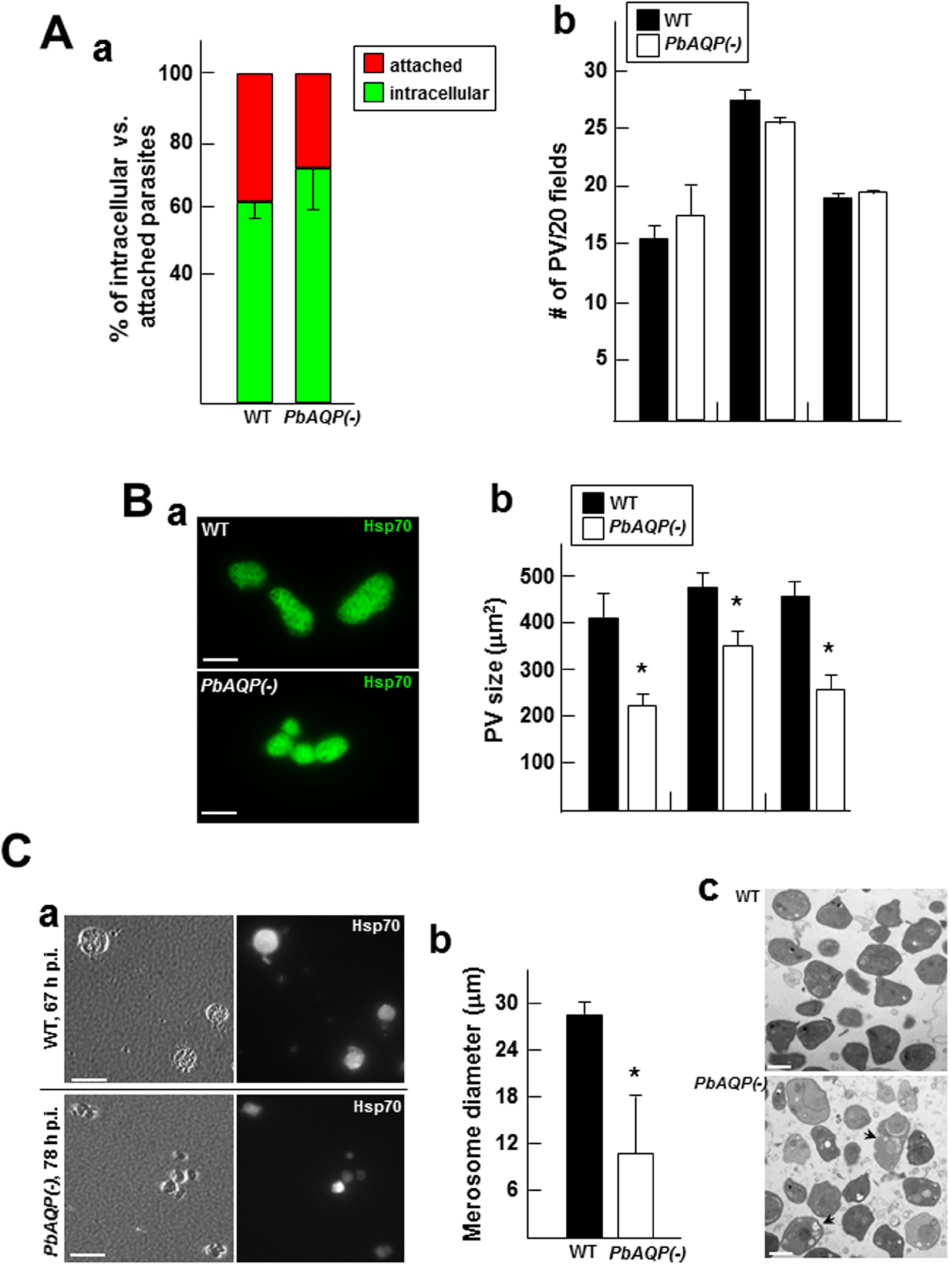
Comparison of the intracellular development of WT and PbAQP-null *P. berghei* in hepatocytes. (**A**) Invasion of Hepa1-6 cells by WT and *PbAQP*(-) sporozoites. Panel a: Hepa1-6 cells were infected with sporozoites for 2 h before fixation and processing for a red-green double IFA using anti-PbCSP antibodies to enumerate intracellular and extracellularly attached parasites. Data expressed in percent of total parasites, are means ± S.D., n = 3 independent assays. Panel b: Hepa1-6 cells were infected for 50 h before fixation and immunostaining using anti-Hsp70 antibodies to count randomly selected PV per 20 microscopic fields. Data are means ± S.D., n = 4 independent assays. (**B**) Replication of WT and PbAQP(-) parasites in Hepa1-6 cells. Panel a: IFA on parasites treated as described in panel b of Fig. 3A. Panel b: quantification of PV size as determined by Volocity on 60–77 PV per parasite strain. Data of PV surface area in μm^2^, are means ± S.D., n = 3 independent assays. *p < 0.05. Bars, 10 μm. (**C**) Merosome production by WT and *PbAQP*(-) parasites. Panel a: Hepa1-6 cells were infected for 67 h (WT parasites) and 78 h (*PbAQP*(-)) before collecting floating merosomes for immunostaining with anti-Hsp70 antibodies and counting in an hemocytometer. Panel b: quantification of the size distribution of merosomes stained for Hsp70, measured by Volocity software. Data of merosome diameters in μm, are means ± S.E.M. from 4 independent infections (n = 18–25 merosomes per parasite strain). *p < 0.05. Bars, 30 μm. Panel c: Transmission EM of hepatic merozoites released from merosomes obtained as described in panel a. Bars, 400 nm.

The developmental delay of PbAQP-null parasites in liver could be attributable to defects in invasion, replication, or both. To examine the potential role of PbAQP in parasite invasion, we performed invasion assays in hepatocytes *in vitro*, utilizing a differential red-green staining assay and antibodies against the sporozoite plasma membrane protein CSP in which extracellular/attached parasites were stained in red while intracellular/invaded parasites fluoresced in green. Hepa1-6 cells were infected with WT or PbAQP-null sporozoites for 2 h, washed, fixed and processed for the double staining assay. No difference in the invasion rate was observed between knockout and control parasites (Fig. 3A, panel a).

In parallel assays, Hepa1-6 cells were infected 50 h before fixation and staining with anti-Hsp70 antibodies to identify and count the PV. The number of PV was similar in cells infected with WT and PbAQP-null parasites (Fig. 3B, panel b), confirming that the lack of PbAQP does not impact parasite invasion and PV formation. However, during our microscopic observations, we noticed that PV containing PbAQP-null parasites were abnormal in shape and appeared smaller than PV of WT parasites, indicating a robust replication defect (Fig. 3B). This phenotype was quantified by measuring the size of the PV for WT and PbAQP-null schizonts using Volocity software. The surface area of the PV with PbAQP-null parasites was approximately 60% of the surface area calculated for WT PV.

### Compared to wild-type parasites, PbAQP-null parasites produce smaller merosomes that appear later during infection

To successfully release infective merozoites into blood vessels, the PV membrane of the schizonts disintegrates, liberating hepatic merozoites in the hepatocyte cytoplasm^12^. Then, these hepatic merozoites bud from the hepatocyte as merosomes that are delimited by the host plasma membrane, enter the bloodstream, and upon rupture of the merosomal membrane, the free hepatic merozoites invade red blood cells. We have shown that PbAQP-null sporozoites injected into mice produced a blood stage infection, though with a significant delay (Fig. 2A), likely due to slower replication in liver cells (Fig. 3B).

We then examined the capability of these knockout parasites to form infectious merosomes. Hepa1-6 cells were infected with WT and PbAQP-null sporozoites and inspected for merosome release. WT merosomes of *P. berghei* are usually apparent, floating in the culture medium from 63 h p.i. As expected, we observed merosomes around 65 h p.i. for control parasites. In contrast, at that time, the cell monolayer infected with PbAQP-null parasites were still largely intact, with very few, small merosomal structures visible in the culture medium. At 78 h p.i., the majority of merosomes of knockout parasites were seen budding from hepatocytes. The number of WT and PbAQP-null merosomes released into culture supernatants at 67 h p.i. were counted using a hemocytometer, and the merosome number was normalized to the number of PV present at 48 h p.i. The number of merosomes expressed as percent of PV corresponded to 62 ± 9% and 8 ± 1%, for WT and knockout parasites respectively, for 4 independent infections (*p* < 0.001). However, at 78 h p.i., the number of PbAQP-null merosomes collected from supernatant reached 59 ± 11%, showing no statistical difference from the number of WT merosomes at 67 h p.i. This suggests that WT and PbAQP-null parasites produce comparable amounts of merosomes but the final production of merosomes was considerably delayed in knockout parasites. Our microscopic observations reveal that PbAQP-null merosomes appeared smaller than WT merosomes (Fig. 3, panel a). Immunostaining of merosomes with anti-Hsp70 antibodies to measure their size using Volocity software shows that the averaged diameter of knockout merosomes was approximately 40% of the mean diameter of WT merososmes, indicating that PbAQP-null merosomes contain less merozoites (Fig. 3C, panel b).

### Liver PbAQP-null merozoites have a delayed prepatent period compared to wild-type merozoites

We performed EM to inspect the ultrastructure of merozoites produced by WT and PbAQP-null parasites. Merozoites were mechanically released from merosomes by passage through syringes. Compared to WT merozoites, many PbAQP-null merozoites were heterogeneous in size, less electron-dense, and contained many unidentifiable organelles (Fig. 3C, panel b, arrow). Given these abnormal morphological features, we hypothesize that PbAQP-null merosomes contain less infectious merozoites than WT, which would explain the delay in the prepatent period observed with knockout sporozoites (Fig. 2A).

**Figure 4:**
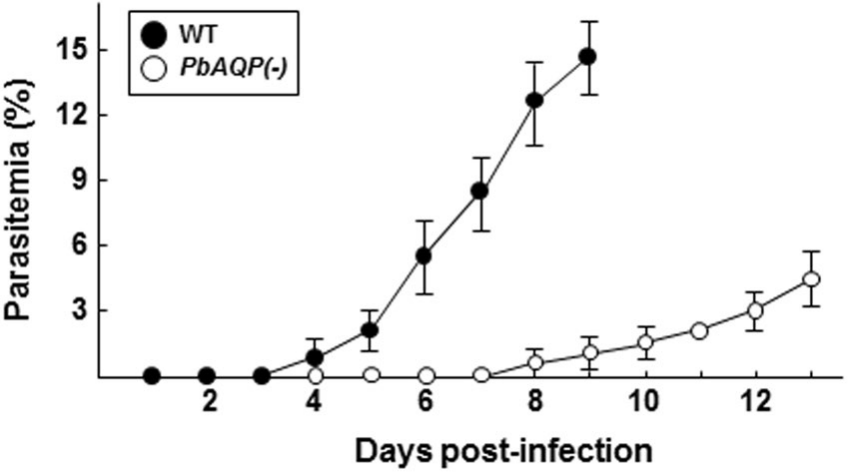
Infectivity of PbAQP-null merosomes. Detection of blood stage parasitemia following injection of merosomes. Five merosomes from WT parasites or 15 from PbAQP-null merosomes were i.v. injected per mouse and parasitemia was monitored daily for 13 days by Giemsa staining on slides. Results from two representative experiments are shown and the average daily parasitemia ± S.D. of 7 mice infected with WT parasites and 8 mice infected with knockout parasites are plotted.

To directly examine the infectivity of PbAQP-null merozoites and ability to invade red blood cells, we collected merosomes from *in vitro* cultures of WT and PbAQP-null parasites and injected them into mice by i.v. As PbAQP-null merozoites are about one third as large as WT, the number of merosomes injected per mouse was 5 and 15 for WT and knockout parasites, respectively. Upon i.v. injection of WT merosomes, mice became positive for blood stage parasites on day 4 after inoculation (Fig. 4A). In contrast, mice injected with PbAQP-null merosomes exhibited a significant delay in the prepatent period, with mice developing detectable parasitemia only on day 8 after injection.

### PbAQP-null merozoites are defective in glycerol uptake for incorporation into phosphatidylcholine and contain less phosphatidylcholine than wild-type merozoites

**Figure 5:**
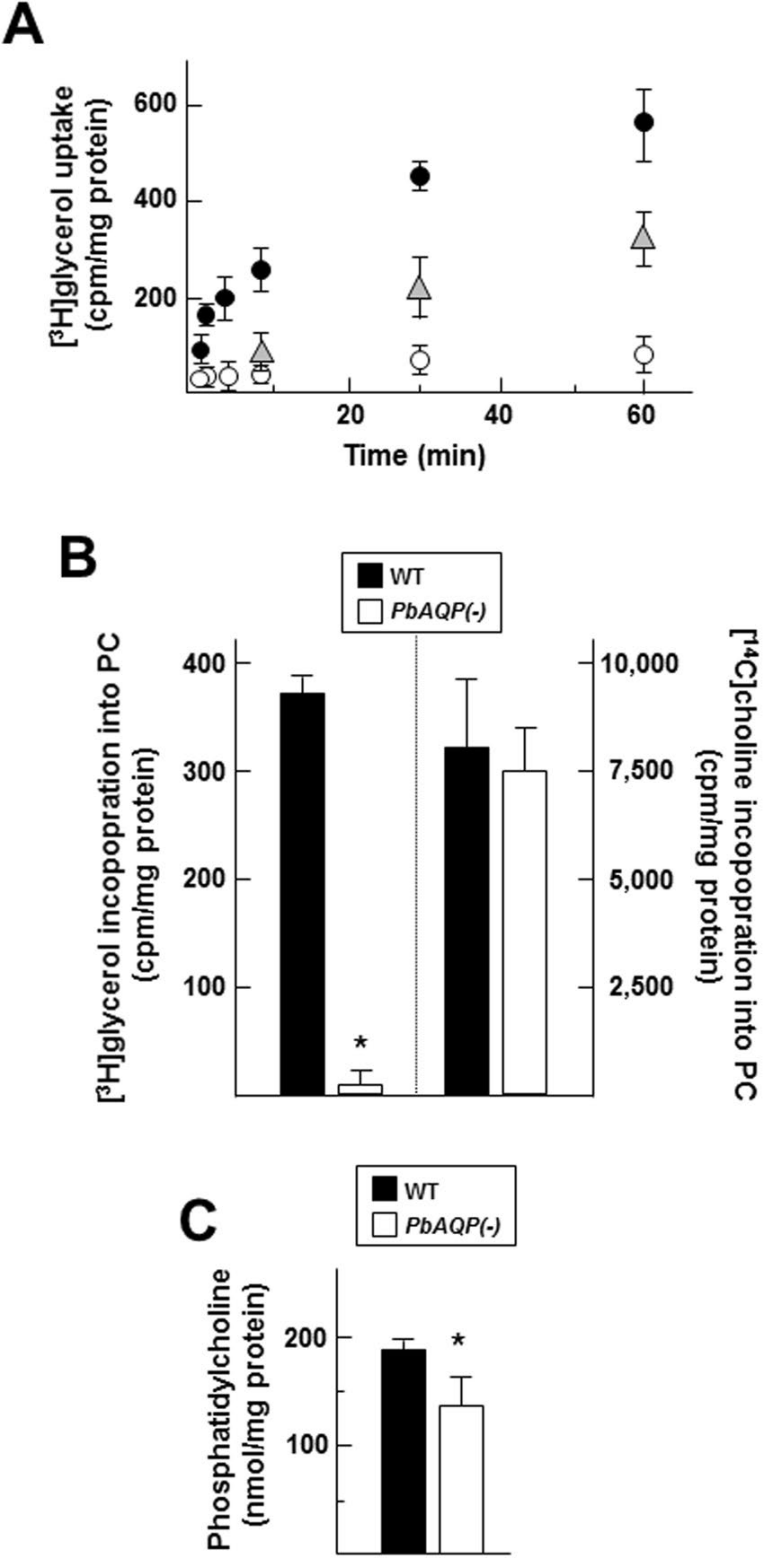
Comparison of glycerol uptake and incorporation into PC between of WT and PbAQP-null merozoites. (**A**) Uptake of [^3^H]glycerol by WT and *PbAQP*(-) hepatic merozoites isolated from merosomes. The uptake of radiolabeled glycerol by hepatic merozoites (close circles for WT and open circles for knockout) was measured after 2, 4, 6, 10, 30 and 60 min incubation at 37 °C, followed by rapid spin through oil and radioactivity determination of the samples. In parallel assays performed on WT merozoites, a 10-times excess of unlabeled glycerol (corresponding to 10 mM) was added to the incubation medium (triangles). Data in cpm normalized to mg protein, are means ± S.D., n = 4 independent assays. (**B**) Incorporation of [^3^H]glycerol and [^14^C]choline into PC of WT and *PbAQP*(-)merozoites. After 4 h incubation at 37°C, parasites were washed, their lipid extracted and run on TLC plates for PC separation. Spots corresponding to PC revealed by iodine vapor were cut and counted for radioactivity. Data in cpm for [^3^H]PC and [^14^C]PC normalized to mg protein, are means ± S.D., n = 3 independent assays. *p < 0.001. (**C**) Quantification of PC in WT and PbAQP(-)merozoites using a commercial enzymatic kit containing phospholipase, choline oxidase, peroxidase, and 4-aminoantipyrin. The quantification of PC was performed after 15 min by measuring the absorption at 492 nm. Values are means ± S.D., n = 3 independent assays. *p < 0.05.

Next, we examined the capability of hepatocyte-derived merozoites to import exogenous glycerol. Merozoites liberated from merosomes were incubated in the presence of [^3^H]glycerol from 2 min to 60 min, and radioactivity counts associated with the parasites were measured at the different time points. An association of radioactive glycerol with WT merozoites increased with incubation time (Fig. 5A). The specificity of glycerol transport in merozoites was assessed by the addition a 10-fold excess of unlabeled glycerol to the incubation mixture. This competitive transport assay shows half the level of radioactive glycerol inside parasites at each time point. Incubation of PbAQP-null merozoites with [^3^H]glycerol resulted in negligible radioactivity associated with knockout parasites, indicating that like PbAQP-null blood forms, hepatic PbAQP-null merozoites display defective transport of glycerol across their plasma membrane.

Next, we examined the fate of exogenous glycerol internalized by hepatic merozoites. A previous study on blood trophozoites of *P. berghei* demonstrated that exogenous glycerol is incorporated into glycerophospholipids such as phosphatidylcholine (PC) and phosphatidylethanolamine^13^. Hepatic WT and PbAQP-null merozoites were incubated with [^3^H]glycerol for 4 h, allowing for the synthesis of phospholipids by these parasites. Lipids were then extracted and separated by thin-layer chromatography (TLC) to isolate PC and the amount of radioactivity incorporated into PC was counted. Radioactive PC was detected in WT parasites, indicating that merozoites utilize glycerol provided in the medium for PC synthesis (Fig. 5B). As expected, almost no radiolabeled PC was identified in PbAQP-null merozoites, indicating a role for PbAQP in glycerol transport for PC formation. Like mammalian cells, *Plasmodium* parasite are choline-auxotroph and express a choline-like carrier to acquire this precursor of PC^14^. As a control for PbAQP-null merozoite viability in our assays, we performed metabolic assays using [^14^C]choline to monitor the formation of [^14^C]PC. No significant differences in radioactivity were associated to the PC spots on TLC plates between WT and PbAQP-null merozoites (Fig. 5B).

Finally, to inspect whether PbAQP makes an important contribution to PC synthesis in merozoites via facilitating glycerol permeation, we isolated hepatic WT and PbAQP-null merozoites to quantify PC concentrations using an enzymatic assay. A moderate but significant reduction in PC amounts was observed in knockout parasites compared to WT parasites (Fig. 5C). This suggests that merozoites use PbAQP as first step for PC biosynthesis. However, despite the absence of exogenous glycerol uptake by PbAQP-null parasites, the knockout parasites seem able to divert glycerol molecules from intraparasitic sources to form sufficient amounts of PC to survive.

## Discussion

Our previous study reported that the single PbAQP gene expressed by *P. berghei* contributes in part to parasite development in red blood cells^5^. In this study, we demonstrated that PbAQP also plays a supportive role in the optimal infection of the liver by the parasite. The significant delay in blood stage infection after sporozoite inoculation into mice suggests that PbAQP functions at one or more steps between the sporozoite’s arrival at the liver and the initiation of the blood stage infection. Our experiments suggest that while PbAQP-null sporozoites invade liver cells normally, intrahepatic parasites form smaller PV and merosomes than WT parasites, indicating intracellular replication defects in the knockout. Additional experiments with mechanically ruptured merosomes also point to decreased infectivity of hepatic merozoites arising from mature liver stage parasites. Together with the increased expression of PbAQP during liver stage development, our observations suggest an important role for PbAQP in preparing hepatic merozoites for infection of red blood cells.

Despite their incompetence in importing glycerol from the environment, hepatocyte-derived merozoites lacking *PbAQP* are nevertheless viable. Surprisingly, these knockout parasites contain PC, although to a lesser extent than WT parasites, which raises the issue of the origin of glycerol necessary for the glycerophospholipid backbone in PbAQP-null parasites. One possibility would be that the knockout parasites retrieve PC intact from host cells. In support to this idea, it has been previously demonstrated that *P. berghei* scavenge and accumulate host PC during their intracellular development in hepatocytes^15^. In hepatocytes lacking choline phosphate cytidyltransferase, the rate-limiting enzyme in the Kennedy pathway for PC synthesis, *P. berghei* infection is greatly reduced.

Another possibility would be that knockout parasites cope with *AQP* deletion by using glycerol or glycerol metabolites produced by their own biochemical pathways. Glycolysis could be a provider of glycerol for the glycerolipid backbone through the reduction of dihydroxyacetone phosphate into glycerol 3-phosphate. However, this reduction step would be detrimental for the parasite because it would create oxidative stresses due to the loss of NADH, resulting in the accumulation of oxidized glutathione that damage membranes. As a matter of fact, examination of the ultrastructure of PbAQP-null merozoites reveals several cytopathies featuring membrane abnormalities that may be due to oxidative damage.

The cellular anomalies PbAQP-null merozoites may also be caused by the accumulation of toxic wastes in the knockout if PbAQP functions in an exit pathway for harmful metabolites. To this point, the AQP of *P. falciparum*, *Toxoplasma gondii* and *Trypanosoma brucei* is permeable to ammonia, facilitating the efflux of this toxic molecule resulting from glutamine deamination^7^. If *P. berghei* is also ammonotelic, PbAQP-null parasites would then be exposed to excess ammonia in the cytosol, leading to superoxide anion radicals and DNA damage in the parasite^16^.

Serum constitutes a rich source of glycerol that accumulates at concentrations ranging between 0.05 and 0.4 mM in humans^17^. Glycerol is a product of triacylglycerol lipolysis occurring in adipose tissues, and is released from adipocytes through AQP7 in the serum. Glycerol is then transported to the liver, which is the main organ for glycerol metabolism^18^. In the liver, hepatocytes express AQP9 to acquire glycerol^19,20^, which serves as a precursor for de novo synthesis of glucose (gluconeogenesis) and glycerolipids^17,21^. Hepatocytes could represent a priori a glycerol profuse environment for *Plasmodium*, however, once internalized via AQP9, glycerol is rapidly and irreversibly phosphorylated by glycerol kinase and glycerol 3-phosphate (G3P) is the most abundant form of glycerol in hepatocytes. Like any aquaglyceroporins, PbAQP is not permeable to negatively charged G3P, making this form of glycerol non-utilizable by intrahepatic parasites. However, glycerol is also endogenously produced by hepatocytes, as a result of the breakdown of glucose, proteins, pyruvate, triacylglycerols and other glycerolipids^22^, and released glycerol from these breakdown products may be intercepted by intrahepatic parasites.

In erythrocytes, glycerol is internalized via AQP9 but these cells lack glycerol kinase or any metabolic pathways employing glycerol, thus making the physiological role of AQP9 in erythrocytes enigmatic^23^. Opportunistically, *P. berghei* infecting red blood cells may take advantage of host AQP9 for glycerol supply from the serum as a slower growth rate in AQP9-null mice was reported. It would be interesting to examine if the intrahepatic development of *Plasmodium* is hampered in AQP9-null mice, even if that exogenous glycerol transiting through AQP9 is converted to G3P and likely unavailable to the parasite.

Host glycerol in hepatocytes must cross the PV membrane to be available to the parasite. This step could be achieved by the passage of glycerol through pores within this membrane, which allow the transit of solutes <850 Da^24^. After internalization into intrahepatic parasites via PbAQP, glycerol may be phosphorylated by a glycerol kinase present in the *P. berghei* genome (PBANKA_1364300.1), as a first step in the biosynthesis of glycerolipids. In *P. falciparum* blood forms, a glycerol kinase (PfGK) activity has been characterized and PfGK is involved in phospholipid synthesis, as demonstrated by the incorporation of radioactive glycerol into PC and phosphatidylethanolamine but ~50% less incorporation of glycerol into these lipids in PfGK-deficient parasites, compared to WT parasites^13^. Like PbAQP-null parasites, *Plasmodium* lacking *PfGK* are viable, showing partial attenuation of their virulence in mice. If *Plasmodium* parasites are largely dependent on exogenous glycerol for phospholipid synthesis, it could be interesting to examine whether *Plasmodium AQP* are co-expressed with the parasite glycerol kinase gene. Future experiments should include the generation of a double knockout strain for *AQP* and *glycerol kinase* to examine the virulence of double knockout parasites and investigate whether the combined effects of these two deletions would result in a significant impairment of phospholipid production.

PC and phosphatidylethanolamine are the two major phospholipids of malaria parasites, accounting for 40 to 50% of PC and 35 to 45% of phosphatidylethanolamine^25,26^. Targeting the glycerophospholipid metabolic pathways for the treatment of malaria has stimulated great interest for four decades, due to the importance of these lipids for the proliferation and pathogenesis of *Plasmodium* parasites^27^. An initial point of interference with phospholipid production would then be *Plasmodium* AQP. Glycerol uptake by *Plasmodium* may be particularly crucial for the parasite upon the limitation of host glucose^28^.

Encouragingly, the PfAQP sequence displays considerable sequence differences from human AQP homologs (~30% identity) and could thus be considered as a valid drug target for several reasons. First, the inner protein core for solute transport through PfAQP is relatively conserved, but the competition of *Plasmodium* with the host for essential solutes would restrict parasite populations from evading the therapeutic pressure of PfAQP inhibitors^29^.

Second, PfAQP exhibits unique structural features in the extracellular connecting loop C, which is part of the selectivity filter for water and solutes, and thus presents a target for specific inhibitors.

Third, PfAQP is very sensitive to the concentration of solutes and can be easily clogged. Studies on the chemical-potential profile of glycerol in PfAQP reveals that when the concentration of glycerol is higher than the dissociation constant of glycerol in PfAQP (corresponding to 14 µM)^30^, PfAQP does not function properly and *P. falciparum* would lose the ability to maintain water homeostasis and excrete metabolic wastes. Molecules that share the same chemical-potential profile of glycerol, e.g., erythritol or sorbitol, could then hold the potential to dwell inside the PfAQP channel at the high μM range, leading to occlusion of the pore^31–33^. Moreover, proton motion analysis of the PfAQP structure shows that the binding pocket of the parasite AQP is deeper and smaller in diameter compared to human AQP, which results in the enhanced binding and strong fitting of inhibitors in PfAQP^34^. *In silico* analysis would help to design targeted molecules that selectively occupy the *Plasmodium* AQP, blocking nutrient and waste passage across the parasite’s plasma membrane.

Fourth, as *Plasmodium* AQP are *per se* channels, they could be exploited as gates of entry for cytotoxic molecules, as demonstrated for arsenous and antimonous acids in *T. brucei* aquaglyceroporins^35^. Aquaglyceroporins are virtually permeable for any linear, uncharged, aliphatic organic molecules smaller than 300 Da^36^. More work dissecting the structural and molecular properties of PfAQP^4^ is imperative to enable the design of solute molecules specifically transported through the malaria parasite AQP.

## Methods

### Reagents and antibodies

All chemicals were obtained from Sigma Chem. Co. (St. Louis, MO) unless indicated otherwise. [^3^H]glycerol (sp. radioactivity: 124 mCi/mmol) and [^14^C]choline chloride (sp. radioactivity: 7.3 mCi/mmol) were purchased from PerkinElmer, Inc (Waltham, MA). Fluorimetric PC assay kit was purchased from Abcam (Cambridge, MA). Primary antibodies used for immunofluorescence assays included rabbit anti-PbAQP (diluted at 1:100)^5^, mouse anti-Hsp70 (diluted at 1:400) from Dr. F. Zavala (Johns Hopkins University) and mouse anti-PbCSP (diluted at 1:200) obtained from MR4 (mAb 3D11; http://www.mr4.org). Anti-IgG antibodies conjugated to Alexa Fluor 350 obtained from Invitrogen (Carlsbad, CA) were used at a dilution of 1:2000. Primers were synthesized by Integrated DNA Technologies (Coralville, IA).

### Mice

Five- to 8- week old female Swiss-Webster were purchased from Taconic (Derwood, MD). All animal procedures were approved by the Institutional Animal Care and Use Committee of the Johns Hopkins University following the National Institutes of Health guidelines for animal housing and care.

### Mammalian cell line, and parasite strains and stages

Mouse Hepa1-6 cells (ATCC CRL-1830) used to host *P. berghei* parasites for transfection studies were obtained from ATCC (Gaithersburg, MD). Cells were grown as monolayers at 37°C in an atmosphere of 5% CO_2_ in α-MEM supplemented with 10% FBS, 2 mM L-glutamine and penicillin/streptomycin (100 U/ml per 100 µg/ml). The *P. berghei* ANKA WT line or PbAQP-null parasites were passaged in *Anopheles stephensi* mosquitoes blood-fed on infected Swiss CD-1/ICR mice as described^5^. To generate *P. berghei* sporozoites, 4–6 days old *A. stephensi* mosquitoes that had been starved for at least 6 h were fed on Swiss-Webster mice infected with *Plasmodium berghei* (ANKA 2.34 strain) or PbAQP-null parasites. Blood fed mosquitoes were incubated at 19°C and 80% relative humidity. At 21–24 days p.i., mosquito salivary glands were dissected in RPMI media and the salivary gland tissue homogenized by passage through a 28 1/2-gauge needle several times to release sporozoites. The sporozoites were counted using a hemocytometer, purified and diluted in RPMI prior to inoculation in Swiss-Webster mice or incubation in the presence of Hepa1-6 cells for various times as described^37,38^. Blood stage parasites were obtained from anesthetized infected mice with a parasitemia above 3% by cardiac puncture. Blood was collected into syringes coated with a stock solution of 25,000 U/ml of heparin to prevent clotting and washed with PBS prior to saponin lysis. Merosomes were harvested from a culture supernatant of Hepa1-6 cells between 61 and 78 h p.i. and hepatic merozoites were collected from merosomes ruptured through a 30-gauge needle and purified as described^39^.

### Transcriptional analysis

RNA was purified from blood stage parasites, sporozoites, *Plasmodium* infected-hepatocytes using the RNeasy RNA purification kit (Qiagen, Valencia, CA). cDNA synthesis was performed using the High capacity cDNA Reverse Transcription Kit (Applied Biosystems, Carlsbad, CA). Equivalent amounts of 100 ng RNA were used as template for each assay. The cDNA generated was mixed with SYBR® Green PCR Master Mix (Applied Biosystems) and specific primers for *PbAQP* and *18s ribosomal-RNA*. Relative expression of *PbAQP* was normalized by the 18S ribosomal-RNA gene for each parasite stage. Amplification was monitored by the 7000 sequence detection system (Applied Biosystems). Primer sequences were for *PbAQP* (sense: 5′-GGGAAGATCTATGAAAGTAACATTTGGTAATGAA-3′ andantisense: 5′-GGGAAGATCTTTATATTTCTAAGGCGCCTTTATC-3′) and for 1*8s* (sense: 5′-AAGCATTAAATAAAGCGAATACATCCTTAC-3′ and antisense: 5′-GGAGATTGGTTTTGACGTTTATGTG-3′). As a negative control, a second sample of −RT cDNA was also generated by omission of the reverse transcriptase and used for PCR. Quantitation of the bands of the PCR-produced fragments was done by drawing ROIs around largest band of the PCR products for each gel and saved in *.zip file to collect numbers that are integrated density of entire box minus background using the ImageJ analysis program.

### Virulence of Plasmodium strains in animals

To assess the virulence of sporozoites, 6 to 9 Swiss-Webster mice were infected via tail vein injection of 1,000 or 10,000 sporozoites (WT or PbAQP-null parasites) per animal in three independent experiments. Parasitemia was monitored daily by Giemsa staining on thin blood smears on glass slides. To assess the relative expression of WT or PbAQP-null parasites in mouse liver, mice were infected with an i.v. injection of 4,500 WT or PbAQP-null sporozoites. Fifty-five h later, livers were collected for RNA extraction, and the total RNA was evaluated for *P. berghei 18S rRNA* levels by reverse transcription followed by real-time PCR, using primers described above. Copy number was ascertained by comparison with a plasmid standard curve.

### Uptake and metabolic labeling experiments

[^3^H]glycerol uptake assays were performed on purified hepatic merozoites using the system described previously^5^. 10E8 merozoites were incubated at 37°C in 1 ml of RPMI containing 4 μCi of tritiated glycerol (25 nmol) and 1 mM of unlabeled glycerol. At various time intervals, ranging between 2 to 60 min, 100 μl of the solution were collected and centrifuged through a dibutyl phthalate layer at 10,000 × g for 50 sec to stop the uptake. The cell pellet was lysed with 0.1% Triton X-100 before scintillation counting. For [^3^H]glycerol or [^14^C]choline incorporation into PC, 3x10E8 merozoites were incubated 4 h at 37°C in 3 ml of RPMI containing 2 μCi of tritiated glycerol (12 nmol) or 10 μCi of radioactive choline (6 nmol), washed and processed for lipid extraction as described^40^. Total phospholipids were resuspended in 150 μl of chloroform:methanol (2:1, v/v) and separated by thin layer chromatography using chloroform:methanol:water (100:42:1; v/v/v) as solvent and run in parallel on silica gel plates with PC as standard identified by iodine vaper, before counting. Values for radioactive PC in the parasites were normalized by parasite protein content.

### Microscopy

Immunofluorescence assays of *Plasmodium*-infected cells were performed as previously described^38^. Briefly, cells were fixed in a solution consisting of 4% paraformaldehyde and 0.02% gluteraldehyde in PBS for 15 min, washed with PBS, permeabilized with 0.3% Triton X-100 for 5 min and washed 3 times with PBS. Samples were blocked with 3% BSA dissolved in PBS for 45 min and probed with primary antibodies diluted in blocking buffer for 1 to 2 h. Samples were then washed 3 times and probed with secondary antibodies diluted in blocking buffer for 45 min. Coverslips were mounted onto glass microscope slides with or without DAPI. Images were acquired on a Nikon Eclipse E800 microscope equipped with a Spot RT CCD Camera and processed using Image-Pro-Plus software (Media Cybernetics, Silver Spring, MD) before assembly using Adobe Photoshop (Adobe Systems, Mountain View, CA). For electron microscopy, merosomes of *P. berghei* ANKA WT line or PbAQP-null parasites were fixed in 2.5% glutaraldehyde (Electron Microscopy Sciences; EMS, Hatfield, PA) in 0.1 M sodium cacodylate buffer (pH 7.4) for 1 h at room temperature, and processed as described^38^ before examination with a Philips CM120 Electron Microscope (Eindhoven, the Netherlands) under 80 kV.

### Phenotypic analysis of liver stage

After immunostaining with anti-Hsp70 antibodies, the size of the PV and merosomes from WT or PbAQP-null parasites was measured 50 h p.i. by determining the area of the parasite at its greatest circumference and quantified as described^38^. For invasion assays, semi-confluent Hepa1-6 cells were infected with WT or PbAQP-null sporozoites for 2 h to allow invasion, washed with PBS and processed for red-green double immunostaining assay using anti-PbCSP antibodies, which distinguishes intracellular from extracellular parasites as described^41^. To determine the size of the PV and merosomes, the perimeter of PV and merosomes was delineated, at their largest circumference based on the fluorescence intensity of the Hsp70 signal, using the ‘region of interest’ tool and the area was calculated using Volocity software.

### Statistical Analysis

*p*-values were determined using a two-tailed end *t*-test for samples with unequal variance.
